# Predictive value of ALBI score in assessing prognosis of NSTEMI: a retrospective cohort study

**DOI:** 10.3389/fcvm.2026.1768037

**Published:** 2026-04-22

**Authors:** Xiaoqiang Chen, Xintao Zhou, Chuanglu Zhao, Lishan Chen, Shuyin Wang, Xinwen Min, Jishun Chen, Xiaolei Li

**Affiliations:** Sinopharm Dongfeng General Hospital (Hubei Research Center of Hypertension), Hubei University of Medicine, Shiyan, Hubei, China

**Keywords:** ALBI, GRACE, mortality, NSTEMI, predicted value

## Abstract

**Objective:**

This study aims to evaluate the predictive value of the albumin-bilirubin (ALBI) score for mortality risk in patients with non-ST-segment elevation myocardial infarction (NSTEMI).

**Methods:**

A retrospective analysis was conducted on 982 patients with NSTEMI. A multivariate Cox proportional hazards model was established to analyze the predictive value of ALBI for mortality after hospital discharge in NSTEMI patients. Additionally, restricted cubic spline (RCS) plots were used to analyze the relationship between ALBI and post-discharge mortality. Operating characteristic (ROC) curves were generated to assess the predictive accuracy of ALBI, and nomograms were constructed to facilitate clinical application in predicting mortality after hospital discharge in patients with NSTEMI.

**Results:**

Among the 982 participants, 62 (6.3%) developed death during the follow-up. In unadjusted Cox regression models, the ALBI index had a hazard ratio (HR) of 5.07 (95% confidence interval CI: 3.13–8.20, *P* < 0.001). In the adjusted models, the relationship still persisted. Furthmore, a non-linear and dose–response relationship between the ALBI index and the primary endpoint was observed (non-linear *P* = 0.050, *P* overall < 0.001). ROC curve analysis revealed good predictive performance for ALBI. The nomogram model correctly separates patients with and without death risk, with an AUC of 0.835. Our model showed improved prediction of death compared to GRACE, or NT-proBNP (all *P* < 0.05).

**Conclusion:**

ALBI was significantly associated with the risk of out-of-hospital death in NSTEMI patients. The novel nomogram based on ALBI, NT-proBNP, and GRACE scores showed an improvement in predicting mortality.

## Background

Acute myocardial infarction (AMI) was a life-threatening condition caused by acute coronary artery occlusion, leading to insufficient blood supply to the corresponding myocardial region and resulting in myocardial necrosis (1). Despite standardized reperfusion therapy and pharmacological treatments significantly improving the prognosis of AMI patients over the past few decades (2, 3), the mortality rate, particularly among elderly patients, remains high (4). Therefore, continuous risk assessment was crucial for identifying high-risk patients and establishing optimal treatment strategies to improve outcomes.

Inflammation (5), nutrition (6) were related to the prognosis of AMI. ALBI, as an emerging indicator, reflects inflammation, oxidative stress and nutrition (7). Since the 2015 study on the association between the ALBI score and liver function assessment and prognosis in hepatocellular carcinoma patients (8), the ALBI score had gradually expanded to research on its correlation with other liver diseases and systemic conditions. Similar reports had also been made in cardiovascular diseases for example, Su et al. (9) conducted a study on 9,749 patients with heart failure,The results showed that the ALBI score could effectively predict the in-hospital mortality rate of patients with heart failure. The ALBI score had been extensively studied in relation to heart failure and cardiomyopathy. A study with an average follow-up of 8.9 years in 4,923 cardiovascular disease (CVD) patients found that higher ALBI scores were associated with increased prevalence of CVD and mortality (10).

The GRACE score was commonly used to assess the risk of non-ST-segment elevation acute coronary syndrome, providing critical decision-making support for clinical diagnosis and treatment (11). However, the GRACE score, which includes factors such as age, blood pressure, heart rate, and creatinine, no longer meets the higher clinical demands for AMI prognosis evaluation. Many researchers had attempted to improve accuracy by combining brain natriuretic peptide (BNP) with GRACE. For instance, Yang et al. (12) demonstrated that combining BNP with the traditional GRACE scoring system yielded higher predictive value for AMI patients than the GRACE score alone. In recent years, the roles of immune, inflammatory and nutritional factors in AMI prognosis have garnered increasing attention. Some scholars (13) had found that inflammation and malnutrition worsen the prognosis of acute coronary syndrome (ACS) patients undergoing percutaneous coronary intervention (PCI), while Other scholars have found that an S-shaped correlation between albumin changes and mortality in elderly AMI patients (14).

However, no studies, domestic or international, have yet reported on the association between the ALBI score and NSTEMI. Therefore, this study aims to retrospectively analyze cases of NSTEMI patients to explore the correlation between ALBI and NSTEMI prognosis. Additionally, it will evaluate the potential improvement of the GRACE score by combining ALBI with NT-proBNP.

## Materials and methods

### Study population

**Figure 1:**
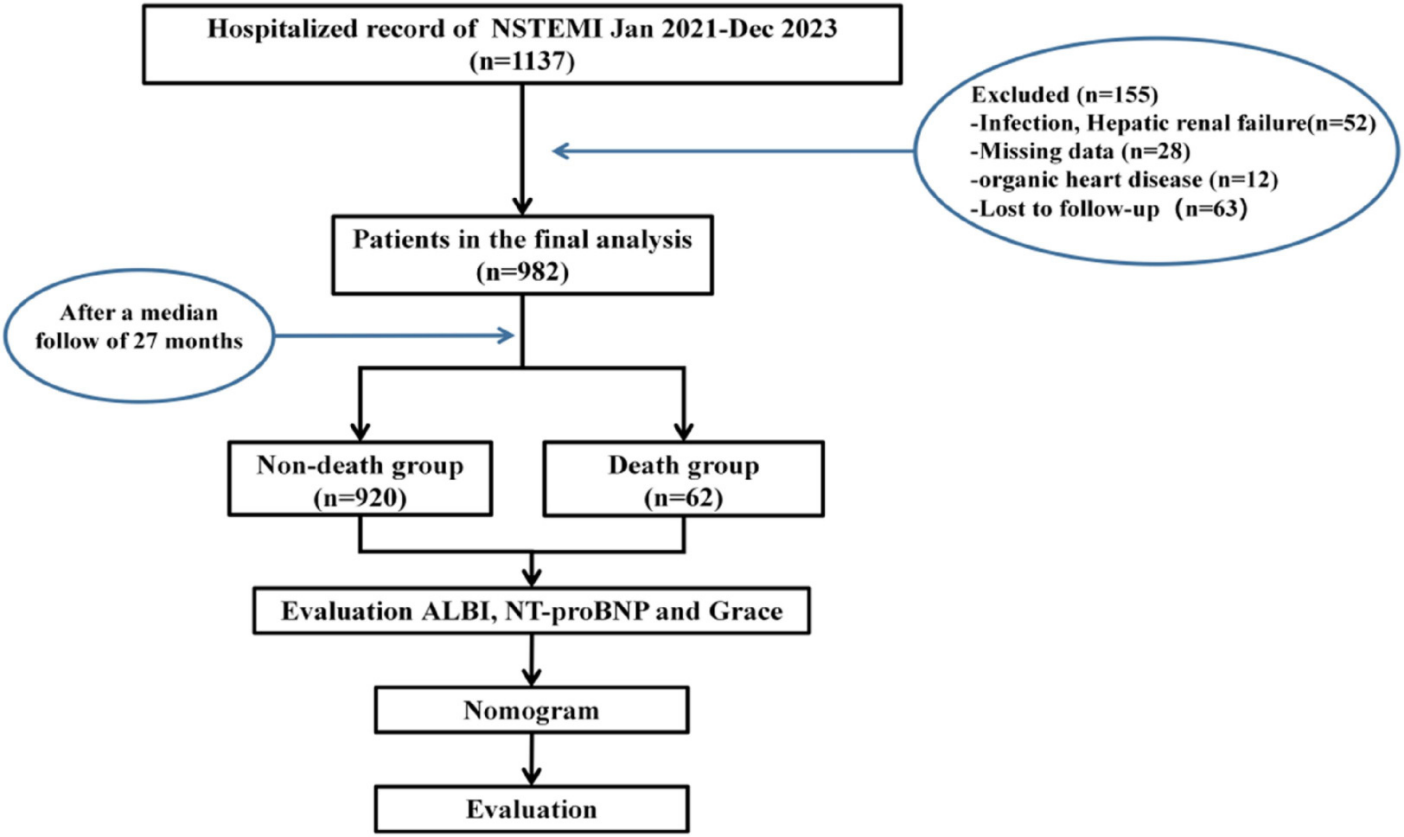
Inclusion of research objects and flow chart.

This study was a single-center, retrospective cohort study. A total of 1,137 hospitalized patients diagnosed with NSTEMI upon discharge from the Cardiac Center of Sinopharm Dongfeng General Hospital affiliated with Hubei University of Medicine between January 2021 and December 2023 were included. These patients were re-evaluated by experienced cardiologists according to inclusion and exclusion criteria, resulting in a final cohort of 982 adult patients (Figure 1).

The inclusion criteria for the study were as follows: (1) Age ≥ 18 years; (2) The diagnosis of AMI was based on the “Fourth Universal Definition of Myocardial Infarction (2018)" (15), The following conditions are considered as acute myocardial infarction: at least one value above the 99th percentile URL and at least one of the following: symptoms of myocardial ischaemia; new ischaemic ECG changes; development of pathological Q waves; imaging evidence of new loss of viable myocardium or new regional wall motion abnormality in a pattern consistent with an ischaemic aetiology; identification of a coronary thrombus by angiography or autopsy. And the exclusion criteria were: (1) Incomplete clinical or coronary angiography data; (2) Post-coronary artery bypass grafting or procedure-related myocardial infarction; (3) Organic heart disease (congenital heart disease, rheumatic heart disease, aortic dissection, severe valvular disease, heart failure); (4) Severe liver or kidney dysfunction (Severe renal insufficiency was defined as eGFR < 30 mL/min. Severe hepatic insufficiency is defined as ALT or AST exceeding the upper limit of normal by a factor of 5, specifically AST > 180 U/L; ALT > 260 U/L), acute infection, malignant tumors, or autoimmune diseases.

## Study methods

### Data collection

We collected patient demographic data using the hospital's electronic medical record system. We had collected clinical data including basic patient information. electrocardiography, left ventricular ejection fraction (LVEF), laboratory examination results and the use of medications.

### Indicator definition

The ALBI score was calculated using the following formula (8): ALBI score = (log_10_ bilirubin × 0.66) + [albumin × (−0.085)], where bilirubin is in μmol/L and albumin in g/L.

The GRACE Score (11, 16) was a risk stratification model for acute coronary syndrome (ACS) developed based on the Global Registry of Acute Coronary Events. It includes eight indicators: age, systolic blood pressure, heart rate, cardiac arrest at admission, Killip class, ST-segment changes, serum creatinine level, and cardiac enzyme changes.

Hypertension (17): Systolic blood pressure (SBP) ≥ 140 mmHg and/or diastolic blood pressure (DBP) ≥ 90 mmHg measured on three different occasions without antihypertensive medication. SBP ≥140 mmHg with DBP <90 mmHg was defined as isolated systolic hypertension. Patients with a history of hypertension currently using antihypertensive medication, even with blood pressure below 140/90 mmHg, were still diagnosed with hypertension. Type 2 diabetes (18) was defined as the use of diabetes medication or self-reported diabetes. Newly diagnosed diabetes was based on diagnostic criteria: diabetes symptoms + random blood glucose ≥11.1 mmol/L or fasting blood glucose ≥7.0 mmol/L or 2 h postprandial blood glucose ≥11.1 mmol/L during an oral glucose tolerance test. Smoking: Continuous or cumulative smoking for more than 6 months, with regular or occasional smoking prior to admission. Alcohol consumption: Drinking alcohol at least once a week before admission.

### Endpoint events and follow-up

Enrolled patients were followed up after discharge. Follow-up information was collected through outpatient visits, telephone contact (obtained from patients or their family members), and review of electronic medical records pertaining to re-hospitalizations. The primary endpoint of this study was all-cause mortality post-discharge; meanwhile, all patients were followed for at least one year or until an endpoint event occurred.

### Statistical methods

Continuous, normally distributed variables were described as mean ± standard deviation and compared with the t-test; non-normally distributed variables were summarized as median (P25,P75) and compared with the Mann–Whitney *U*-test. Categorical variables were expressed as frequency (percentage) and compared using the Chi-square test or Fisher's exact test, as appropriate.

Kaplan–Meier method was applied to construct cumulative survival curves, and differences between groups were compared using the Log-Rank test. Univariate and multivariate Cox proportional hazards models were used to examine the correlation between ALBI and mortality incidence. Restricted cubic splines (RCS) were employed to reflect the dose-response relationship between ALBI and major endpoint events. Receiver operating characteristic (ROC) curves were plotted to indicate the predictive value of ALBI score for mortality.The variance inflation factor (VIF) is calculated to assess multicollinearity among model variables, with variables exhibiting a VIF greater than 5 being excluded. And evaluate the overall model performance using the likelihood ratio test and report the concordance index (C-index) to quantify the model's discriminatory ability.

This study employed the Statistical software used includes SPSS 26.0 and R language 4.1. All tests were twosided, and a significance level of *P* < 0.05 was considered statistically significant.

## Results

### Baseline characteristics

During the follow-up period (median, 27 months), the incidence of death among 982 participants was 62 (6.31%). As shown in Table 1, the study population was stratified into a survival group (*n* = 920) and a death group (*n* = 62).Compared with the survival group, the death group had older age, higher proportion of males, higher GRACE Risk Score and higher proportion of combined diabetes history, and lower body mass index (BMI). Comparisons of pulse rate, LVEF, white blood cell count (WBC), hemoglobin (Hb), K^+^, Na^+^, Urea, creatinine(Crea), glucose (GLU), albumin (ALB), aspartate aminotransferase (AST) and N-terminal pro-B-type natriuretic peptide (NT-proBNP) between the two groups revealed statistically significant differences(*P* < 0.05). Notably, the death group exhibited a lower proportion of patients using aspirin,stains, P2Y12 receptor blockers, angiotensin converting enzyme inhibitor(ACEI) and β-blockers (*P* < 0.05). Moreover, the comparison of ALBI scores between the survival group and the death group showed a statistically significant difference (−2.38 vs. −2.12, *P* < 0.001).

**Table 1:** Baseline characteristics of different prognostic groups.

Variables	Total (*n* = 982)	Survival (*n* = 920)	Death (*n* = 62)	*P*
Age, (years)	68.00 (57.00, 75.75)	67.00 (57.00, 75.00)	75.50 (67.50, 83.00)	<0.001
Male, [*n*(%)]	683 (69.55)	647 (70.33)	36 (58.06)	0.042
Smoking, [*n*(%)]	444 (45.21)	423 (45.98)	21 (33.87)	0.064
Drinking, [*n*(%)]	444 (45.21)	423 (45.98)	21 (33.87)	0.083
Hypertension, [*n*(%)]	582 (59.27)	544 (59.13)	38 (61.29)	0.738
Diabetes, [*n*(%)]	235 (23.93)	208 (22.61)	27 (43.55)	<0.001
Stroke, [*n*(%)]	53 (5.40)	49 (5.33)	4 (6.45)	0.929
BMI, (kg/m^2^)	23.44 (21.20, 24.61)	23.44 (21.20, 24.61)	21.89 (21.11, 23.89)	0.005
SBP, (mmHg)	132.00 (116.00, 148.00)	132.00 (117.00, 148.00)	128.00 (107.00, 144.75)	0.135
DBP, (mmHg)	77.00 (69.00, 88.75)	77.00 (69.75, 89.00)	75.50 (66.00, 85.75)	0.098
P, (times/minutes)	78.00 (69.00, 90.00)	78.00 (68.00, 90.00)	86.00 (76.00, 102.75)	<0.001
LVEF, (%)	58.00 (48.00, 62.00)	58.00 (50.00, 63.00)	44.00 (37.25, 57.50)	<0.001
WBC, (10^9^/L)	7.86 (6.19, 10.02)	7.80 (6.19, 9.91)	9.19 (6.45, 11.86)	0.036
Hb, (g/L)	134.00 (119.00, 147.00)	136.00 (120.00, 148.00)	116.50 (91.00, 129.50)	<0.001
K^+^, (mmol/L)	3.80 (3.50, 4.10)	3.70 (3.50, 4.05)	4.10 (3.73, 4.38)	<0.001
Na^+^, (mmol/L)	139.00 (137.00, 141.00)	139.00 (137.00, 141.00)	138.00 (135.00, 141.00)	0.043
Urea, (mmol/L)	5.66 (4.47, 7.44)	5.58 (4.41, 7.16)	9.46 (6.38, 13.21)	<0.001
Crea, (umol/L)	73.00 (62.00, 98.75)	73.00 (62.00, 93.00)	119.50 (77.00, 171.00)	<0.001
Glu, (mmol/L)	7.30 (5.90, 9.60)	7.20 (5.90, 9.40)	8.85 (6.57, 11.94)	0.002
ALB, (g/L)	39.00 (36.00, 42.00)	39.00 (36.00, 42.00)	35.00 (32.25, 38.00)	<0.001
ALT, (U/L)	23.50 (16.00, 36.00)	24.00 (16.00, 36.00)	23.00 (15.00, 48.00)	0.500
AST, (U/L)	39.00 (28.00, 65.75)	38.00 (28.00, 62.00)	52.00 (34.25, 121.00)	0.001
TBIL, (umol/L)	13.60 (10.50, 17.90)	13.60 (10.60, 17.80)	12.55 (8.83, 18.93)	0.178
TC, (mmol/L)	4.03 (3.24, 4.90)	4.03 (3.23, 4.87)	3.99 (3.27, 5.35)	0.347
TG, (mmol/L)	1.38 (0.94, 2.09)	1.37 (0.93, 2.10)	1.45 (1.06, 2.06)	0.553
HDL-C, (mmol/L)	0.94 (0.78, 1.12)	0.94 (0.78, 1.13)	0.85 (0.76, 1.04)	0.054
LDL-C, (mmol/L)	2.24 (1.65, 2.98)	2.23 (1.66, 2.96)	2.32 (1.63, 3.35)	0.295
Hs-TnI, (pg/mL)	3,442.15 (634.55, 30,383.05)	3,326.20 (623.00, 30,585.92)	6,295.35 (889.98, 24,194.30)	0.377
NT-proBNP, (pg/mL)	1,097.50 (305.75, 3,444.75)	962.60 (279.00, 2,892.50)	7,035.50 (2,953.50, 11,669.00)	<0.001
GRACE	119.00 (97.00, 150.75)	117.00 (95.00, 146.00)	177.00 (145.50, 196.75)	<0.001
ALBI	−2.36 (−2.60, −2.09)	−2.38 (−2.62, −2.11)	−2.12 (−2.29, −1.84)	<0.001
P2Y12receptor, [*n*(%)]	865 (88.09)	832 (90.43)	33 (53.23)	<0.001
Aspirin, [*n*(%)]	838 (85.34)	807 (87.72)	31 (50.00)	<0.001
Statins, [*n*(%)]	897 (91.34)	859 (93.37)	38 (61.29)	<0.001
ACEI, [*n*(%)]	597 (60.79)	575 (62.50)	22 (35.48)	<0.001
*β*-blockers, [*n*(%)]	630 (64.15)	610 (66.30)	20 (32.26)	<0.001
Diuretics, [*n*(%)]	308 (31.36)	287 (31.20)	21 (33.87)	0.660

BMI, body mass index; SBP, systolic blood pressure; DBP, diastolic blood pressure; P, pulse; LVEF, left ventricular ejection fraction; WBC, white blood cell; Hb, hemoglobin; K, potassium; Na, sodium; Urea, blood urea nitrogen; Crea, creatinine; NA, blood sodium; K, blood urea nitrogen; Scr serum creatinine; GLU, blood glucose; ALB, albumin; ALT, alanine aminotransferase; AST, aspartate aminotransferase; TBIL, total bilirubin; TC, total cholesterol; TG, triglyceride; HDLC, high-density lipoprotein cholesterol; LDLC, low-density lipoprotein cholesterol; Hs-TnI, high-sensitivity troponin I; NT-pro-BNP, N-terminal pro-B-type natriuretic peptide; ALBI, albumin-bilirubin sore; ACEI, angiotensin-converting enzyme inhibitor;.

### Baseline characteristics of groups with different ALBI score levels

Patients with acute myocardial infarction were divided into four groups based on the quartile range of serum ALBI score: (I) Q1 (ALBI score from <−2.6); (II) Q2 (ALBI score from −2.6 to −2.36); (III) Q3 (ALBI score from −2.36 to −2.08); and (IV) Q4 (ALBI score > −2.08).The high ALBI score group showed statistically significant differences compared to the low ALBI score group in terms of age, smoking, BMI, SBP, DBP, GRACE score, LVEF, WBC, hemoglobin (Hb) level, platelet count (PLT), glycated hemoglobin, CRP, serum sodium level, serum calcium level, Crea, Urea, indirect bilirubin(IBIL), ALB, ALT, AST, total bilirubin (TBIL), direct bilirubin (DBIL), cholesterol (TC), triglycerides (TG), low-density lipoprotein cholesterol (LDL-C), and NT-proBNP levels. Moreover, the high ALBI score group exhibited higher usage of aspirin and diuretics compared to the low ALBI score group. Furthermore, the difference between the high and low ALBI score groups was statistically significant with *P* < 0.001. Table 2 shows the patient characteristics according to the ALBI score quartiles.

**Table 2:** Baseline characteristics of the population in different ALBI level group.

Variables	Total (*n* = 982)	Q1 (*n* = 245) (≤−2.60)	Q2 (*n* = 246) (−2.60 to −2.36)	Q3 (*n* = 245) (−2.36 to −2.08)	Q4 (*n* = 246) (>−2.08)	*χ*²	*P*
Age, (years)	68.00 (57.00, 75.75)	58.00 (51.00,70.00)	66.00 (58.00,75.00)	70.00 (61.00,76.00)	72.00 (62.00,78.00)	86.397	<0.001
Male, *n*(%)	683 (69.55)	156 (63.42)	176 (71.84)	171 (69.51)	180 (73.47)	6.755	0.080
Smoking, *n*(%)	444 (45.21)	94 (38.21)	109 (44.49)	112 (45.53)	129 (52.65)	10.405	0.015
Drinking, *n*(%)	175 (17.82)	37 (15.04)	44 (17.96)	49 (19.92)	45 (18.37)	2.091	0.554
Hypertension, *n*(%)	582 (59.27)	135 (54.88)	153 (62.45)	156 (63.42)	138 (56.33)	5.621	0.132
Diabetes, *n*(%)	235 (23.93)	64 (26.02)	64 (26.12)	50 (20.33)	57 (23.27)	3.051	0.384
Stroke, *n*(%)	53 (5.40)	13 (5.29)	17 (6.94)	10 (4.07)	13 (5.31)	2.005	0.571
BMI, (kg/m^2^)	23.44 (21.20, 24.61)	23.44 (21.20,24.61)	23.44 (21.58,24.61)	22.89 (21.20,24.04)	23.25 (21.20,24.04)	12.980	0.005
SBP, (mmHg)	132.00 (116.00, 148.00)	136.00 (121.00,151.00)	132.00 (118.00,148.00)	130.00 (116.000,146.00)	130.00 (110.25,144.75)	13.218	0.004
DBP, (mmHg)	77.00 (69.00, 88.75)	79.00 (72.00,92.00)	78.00 (71.00,90.00)	76.00 (70.00,86.00)	75.00 (66.00,85.00)	19.881	<0.001
P, (times/minutes)	78.00 (69.00, 90.00)	79.00 (70.00,92.00)	77.00 (69.00,88.00)	77.00 (66.000,89.00)	80.00 (69.000,92.00)	3.040	0.386
LVEF, (%)	58.00 (48.00, 62.00)	60.00 (53.00,65.00)	58.00 (50.00,62.75)	57.00 (48.00,62.00)	54.00 (45.00,60.00)	34.919	<0.001
WBC, (10^9^/L)	7.86 (6.19, 10.02)	8.55 (6.60,10.91)	7.45 (6.15,9.47)	7.38 (5.94,9.62)	7.88 (6.08,9.84)	17.199	<0.001
Hb, (g/L)	134.00 (119.00, 147.00)	142.00 (128.00,152.00)	138.00 (123.25,149.00)	130.00 (118.00,142.00)	125.00 (108.25,140.00)	66.631	<0.001
PLT, (g/L)	200.00 (161.00, 242.75)	214.00 (174.00,262.00)	205.00 (164.50,243.75)	194.00 (163.00,234.00)	181.00 (138.25,240.00)	30.272	<.001
HbA1c, (%)	5.90 (5.50, 6.70)	6.20 (5.58,7.00)	5.90 (5.50,6.43)	6.00 (5.60,6.90)	5.85 (5.50,6.60)	8.223	0.042
CRP, (mg/L)	5.01 (1.31, 18.31)	2.52 (0.96,10.77)	3.64 (1.172,11.85)	5.16 (1.22,19.55)	13.69 (3.22,43.07)	81.085	<0.001
K^+^, (mmol/L)	3.80 (3.50, 4.10)	3.80 (3.50,4.10)	3.80 (3.50,4.00)	3.80 (3.50,4.10)	3.70 (3.50,4.20)	2.140	0.544
Na^+^, (mmol/L)	139.00 (137.00, 141.00)	140.00 (138.00,141.00)	139.00 (137.00,141.00)	139.00 (136.00,141.00)	138.00 (136.00,140.00)	20.094	<0.001
Cl^−^, (mmol/L)	107.00 (104.00, 109.00)	107.00 (105.00,109.00)	107.00 (104.00,109.75)	107.00 (105.00,109.00)	107.00 (104.00,109.00)	3.622	0.305
Ca^2+^, (mmol/L)	2.20 (2.12, 2.28)	2.27 (2.20,2.35)	2.22 (2.15,2.29)	2.17 (2.10,2.24)	2.14 (2.07,2.22)	139.394	<0.001
Urea, (mmol/L)	5.66 (4.47, 7.44)	5.25 (4.32,6.79)	5.54 (4.55,7.03)	5.93 (4.71,7.73)	6.11 (4.50,9.21)	19.028	<0.001
Crea, (umol/L)	73.00 (62.00, 98.75)	70.00 (61.00,85.00)	75.00 (62.25,99.00)	74.00 (63.00,101.00)	75.00 (62.00,110.00)	8.158	0.043
Glu, (mmol/L)	7.30 (5.90, 9.60)	7.40 (6.20,9.80)	7.10 (5.90,9.00)	7.50 (6.00,10.60)	7.10 (5.90,9.78)	3.446	0.328
IBIL, (umol/L)	9.00 (6.70, 12.00)	7.80 (5.80,10.30)	8.90 (6.90,11.48)	9.40 (6.60,13.50)	10.75 (7.80,14.98)	66.100	<0.001
ALB, (g/L)	39.00 (36.00, 42.00)	43.00 (42.00,44.00)	40.00 (39.00,41.00)	38.00 (36.00,39.00)	34.00 (33.00,36.00)	700.551	<0.001
ALT (U/L)	23.50 (16.00, 36.00)	26.00 (18.00,36.00)	22.00 (15.25,32.00)	21.00 (16.00,36.00)	27.00 (17.00,43.75)	19.246	<0.001
AST, (U/L)	39.00 (28.00, 65.75)	36.00 (28.00,55.00)	37.00 (28.00,56.00)	38.00 (26.00,68.00)	47.00 (31.00,105.00)	28.870	<0.001
TBIL, (umol/L)	13.60 (10.50, 17.90)	11.40 (8.90,14.50)	13.15 (10.50,16.48)	14.30 (10.70,18.90)	16.50 (12.30,24.00)	112.312	<0.001
DBIL, (umol/L)	4.30 (3.40, 5.70)	3.60 (2.90,4.50)	4.10 (3.30,5.20)	4.70 (3.60,5.90)	5.60 (4.10,7.58)	157.585	<0.001
TC, (mmol/L)	4.03 (3.24, 4.90)	4.37 (3.47,5.24)	4.22 (3.32,5.06)	3.91 (3.29,4.61)	3.63 (3.05,4.42)	41.289	<0.001
TG, (mmol/L)	1.38 (0.94, 2.09)	1.72 (1.07,2.78)	1.46 (0.99,2.16)	1.27 (0.93,1.82)	1.12 (0.82,1.66)	54.504	<0.001
HDLC, (mmol/L)	0.94 (0.78, 1.12)	0.96 (0.79,1.13)	0.92 (0.78,1.10)	0.94 (0.77,1.14)	0.92 (0.77,1.14)	2.605	0.457
LDLC, (mmol/L)	2.24 (1.65, 2.98)	2.41 (1.79,3.11)	2.41 (1.71,3.08)	2.21 (1.64,2.82)	2.05 (1.52,2.72)	20.200	<0.001
Hs-TnI, (pg/mL)	3,442.15 (634.55, 30,383.05)	1,870.80 (606.60, 25,473.90)	3,479.25 (563.8830543.28)	4,242.50 (630.40,30,156.40)	4,420.90 (772.38,3,239.400)	3.752	0.290
NT-proBNP (pg/mL)	1,097.50 (305.75, 3,444.75)	560.00 (167.10,1,655.00)	909.80 (241.93,2,586.75)	1,183.00 (333.70,3,870.00)	2,504.00 (779.10,7264.50)	79.449	<0.001
GRACE	119.00 (97.00, 150.75)	103.00 (82.00,131.00)	112.00 (95.00,144.50)	127.00 (104.00,152.00)	136.00 (110.00,167.00)	100.403	<0.001
ALBI	−2.36 (−2.60, −2.09)	−2.76 (−2.90,−2.69)	−2.47 (−2.54,−2.42)	−2.23 (−2.30,−2.15)	−1.93 (−2.02,−1.78)	919.689	<0.001
Aspirin, *n* (%)	838 (85.34)	190 (77.24)	210 (85.71)	214 (86.99)	224 (91.43)	20.733	<0.001
Statins, *n* (%)	897 (91.34)	216 (87.81)	223 (91.02)	232 (94.31)	226 (92.25)	6.916	0.075
ACEI, *n* (%)	597 (60.79)	139 (56.50)	149 (60.82)	154 (62.60)	155 (63.27)	2.865	0.413
β-blockers, *n* (%)	630 (64.16)	147 (59.76)	151 (61.63)	160 (65.04)	172 (70.20)	6.730	0.081
Diuretics, n (%)	308 (31.37)	101 (41.06)	79 (32.25)	70 (28.46)	58 (23.67)	18.523	<0.001

PLT, platelet; HbA1c, glycated hemoglobin A1c; CRP, C-reactive protein; IBIL, indirect bilirubin; DBIL, direct bilirubin.

### Individual effects of ALBI on outcomes

Multivariate Cox proportional hazards model analysis showed that ALBI was a risk factor for out-of-hospital death in AMI patients, whether as a continuous variable (HR: 3.77, 95% CI: 1.89–7.53, *P* < 0.001) or a categorical variable (HR = 5.04, 95% CI: 1.50–16.88, *P* = 0.009). After adjusting for multiple models by separately incorporating gender, age, blood pressure, medication history, and laboratory indicators, ALBI remains significantly associated with the risk of out-of-hospital death in AMI patients. Details are shown in Table 3. We aslo performed the requested sensitivity analysis by constructing a more parsimonious multivariate Cox proportional hazards model. We adjusted for key clinical covariates, including age, sex, NT-proBNP, and ALBI (see Supplementary Table S1). The hazard ratio for ALBI remained statistically significant and directionally consistent with our main findings, demonstrating the robustness of the association even in a model with fewer covariates.

**Table 3:** Cox regression analysis of ALBI score for predicting out-of-hospital mortality.

Variables	Model1		Model2		Model3		Model4	
	HR (95% CI)	*P*	HR (95% CI)	*P*	HR (95% CI)	*P*	HR (95% CI)	*P*
ALBI (continuous)	5.07 (3.13–8.20)	<0.001	5.30 (3.05–9.21)	<0.001	3.76 (1.88–7.52)	<0.001	3.77 (1.89–7.53)	<0.001
ALBI (categorical)								
Q1 (≤−2.60)	Reference		Reference		Reference		Reference	
Q2 (−2.60 to −2.36)	3.03 (0.82–11.20)	0.096	3.17 (0.86–11.72)	0.084	2.34 (0.61–8.91)	0.213	2.28 (0.59–8.72)	0.230
Q3 (−2.36 to −2.08)	6.89 (2.05–23.20)	0.002	6.28 (1.86–21.19)	0.003	4.50 (1.32–15.34)	0.016	4.39 (1.28–15.00)	0.018
Q4 (>−2.08)	10.67 (3.26–34.96)	<0.001	9.24 (2.81–30.40)	<0.001	4.99 (1.49–16.71)	0.009	5.04 (1.50–16.88)	0.009

HR, hazard ratio; CI, confidence interval.

Model1: Crude.

Model 2: Adjust: gender, Smoking, Drinking, hypertension, diabetes, stroke, BMI, SBP, DBP.

Model3: Adjust: gender, Smoking, Drinking, hypertension, diabetes, stroke, aspirin, statins, ACEI, B-blockers, Diuretics, BMI, SBP, DBP, EF, Urea, Crea, ALT, AST.

Model4: Adjust: gender, Smoking, Drinking, hypertension, diabetes, stroke, aspirin, statins, ACEI, B-blockers, Diuretics, BMI, SBP, DBP, EF, Urea, Crea, ALT, AST, hs-TnI, NT-pro-BNP.

## Evaluation of the impact of the ALBI score on prognosis

### Kaplan–Meier analysis

**Figure 2:**
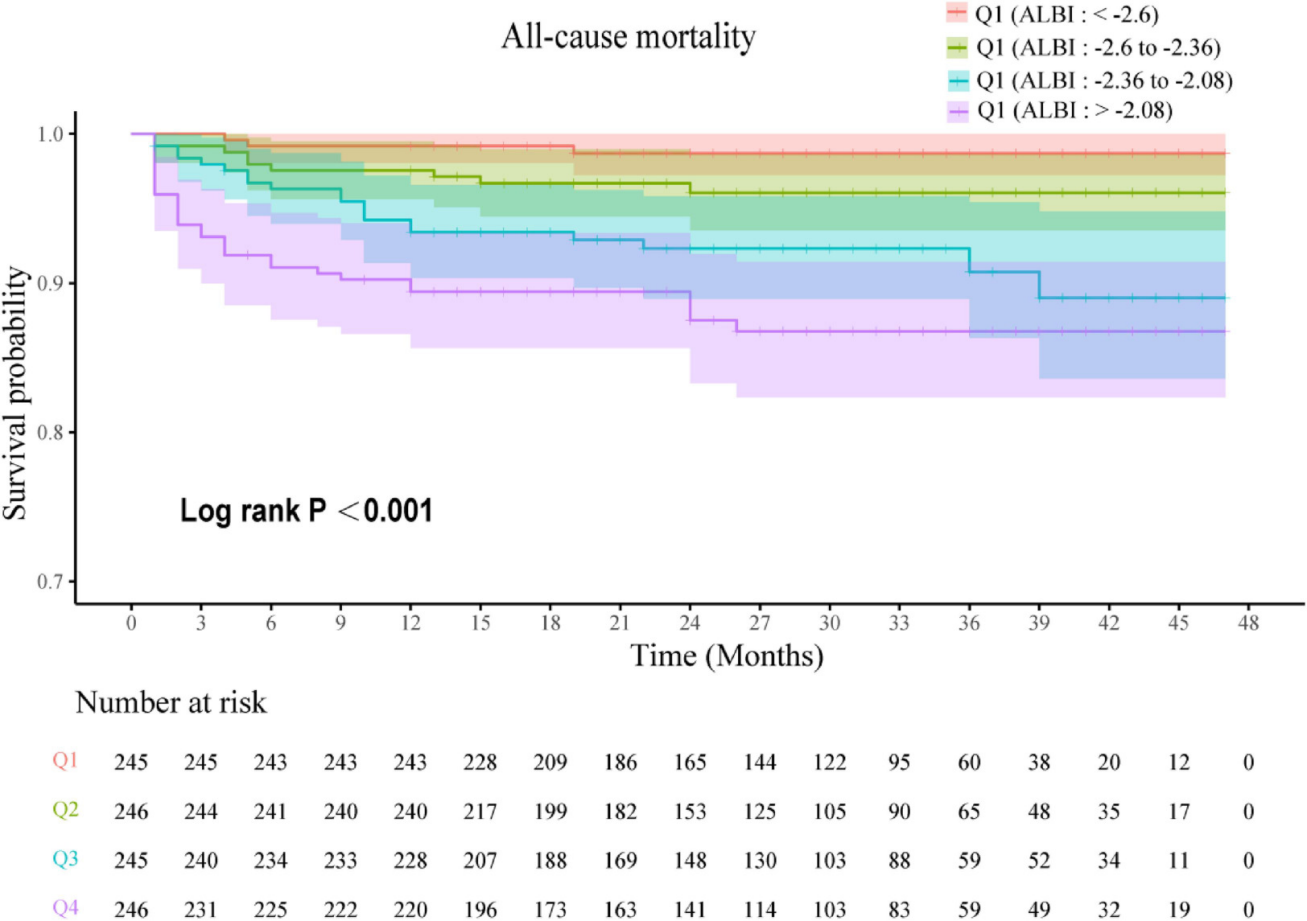
Kaplan–Meier survival analysis curve.

The Kaplan–Meier survival curves (Figure 2) show that patients with ALBI >−2.08 experienced significantly lower survival rates compared to those with ALBI <−2.6 (Log-rank test, *P* < 0.001).

Q1 (ALBI score from <−2.6); Q2 (ALBI score from −2.6 to −2.36); Q3 (ALBI score from −2.36 to −2.08); Q4 (ALBI score > −2.08).

### ROC curve analysis

**Figure 3:**
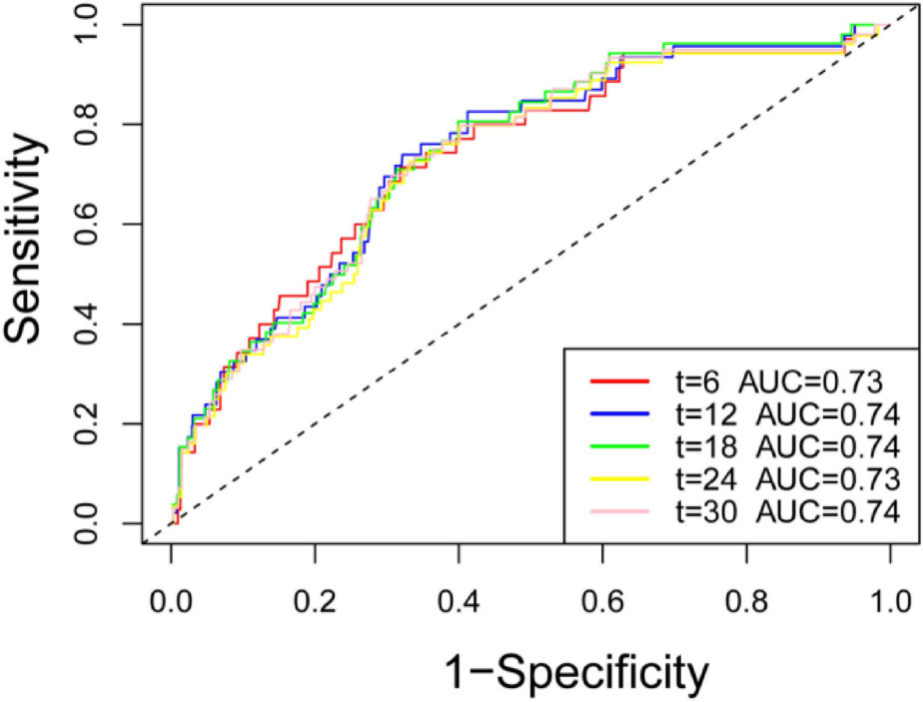
ROC curve of ALBI score for predicting mortality in patients with myocardial infarction.

**Figure 4:**
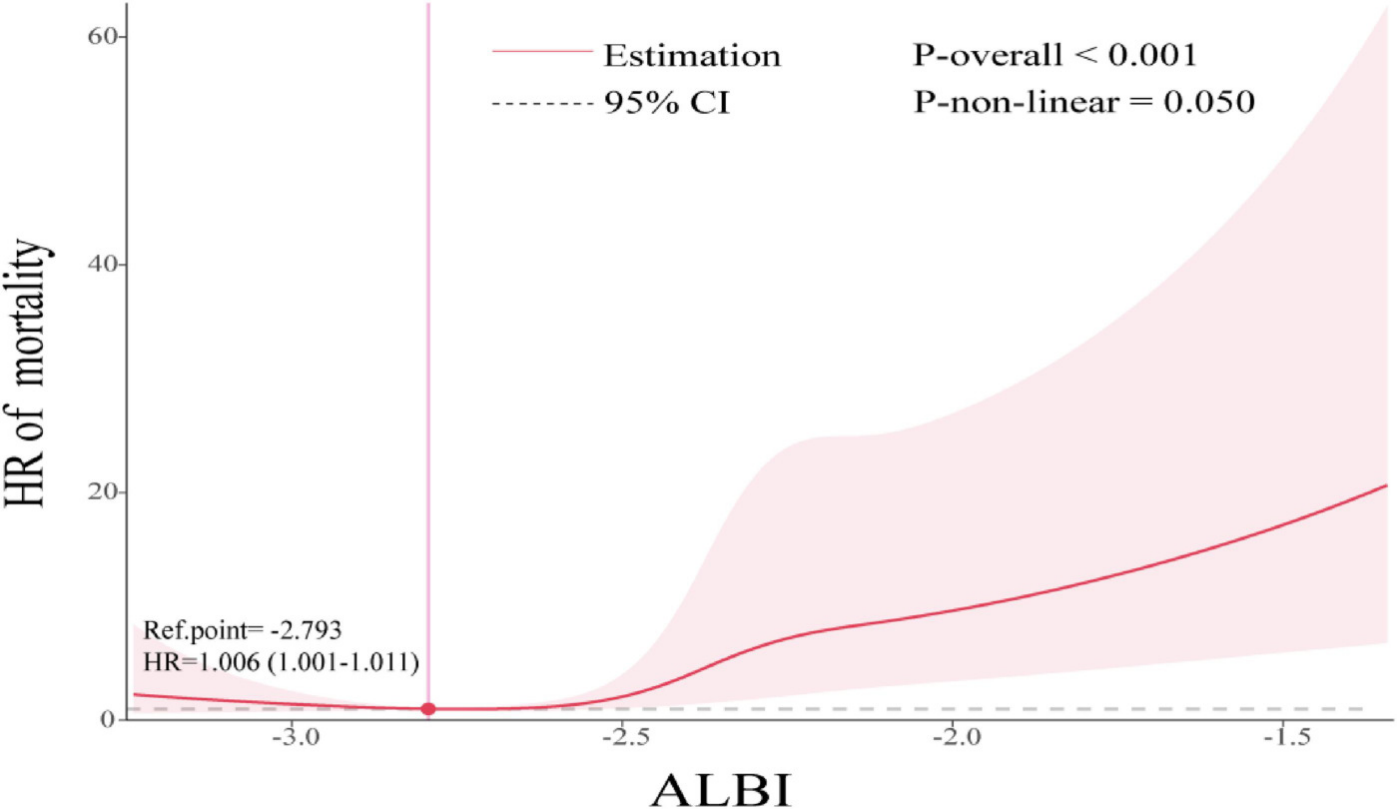
Restricted cubic spline plots based on ALBI score levels.

ROC curves comparing the predictive performance of ALBI for mortality are presented in Figures 3, 4. The results show that the ALBI score has a good predictive value for the mortality of patients with acute myocardial infarction outside the hospital. ALBI achieved an area under the curve of 0.73.

### Restricted cubic spline analysis

The association of the ALBI index with the mortality outcomes was visualized in Figure 3 using restricted cubic spline (RCS). A non-linear and dose–response relationship between the ALBI index and the primary endpoint was observed (non-linear *P* = 0.050, *P* overall < 0.001). Moreover the ALBI index at −2.793 was indicated to be the reference value for the dose–response relationship that participants with a ALBI index higher than −2.793 were associated with higher risk for death (HR = 1.006, 95% CI: 1.001–1.011).

### Construction of nomogram

**Figure 5:**
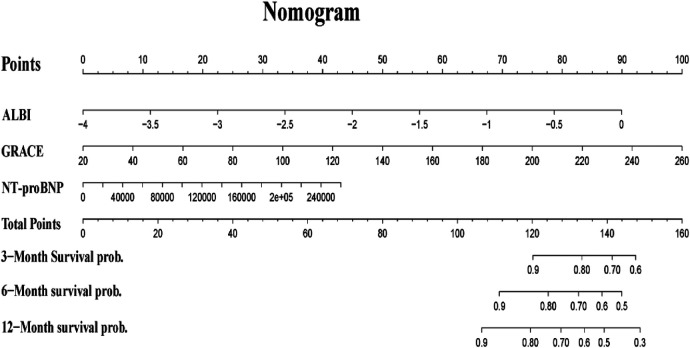
Predictive nomogram of 3-, 6-, and 12-month mortality, in which the total score corresponds to a death probability at the bottom, summing each value of the variable.

To better predict the mortality risk of AMI, we combined ALBI, GRACE scores, and NT-pro BNP to construct a novel nomogram model for easier application in clinical practice, as shown in the Figure 5. The scores corresponding to each predictor variable in the nomogram are summed, and the resulting probability value corresponding to the total score is the probability of risk of death.

### The predictive value of the ALBI score combined with the GRACE score and NT-proBNP for the mortality of patients with myocardial infarction

**Figure 6:**
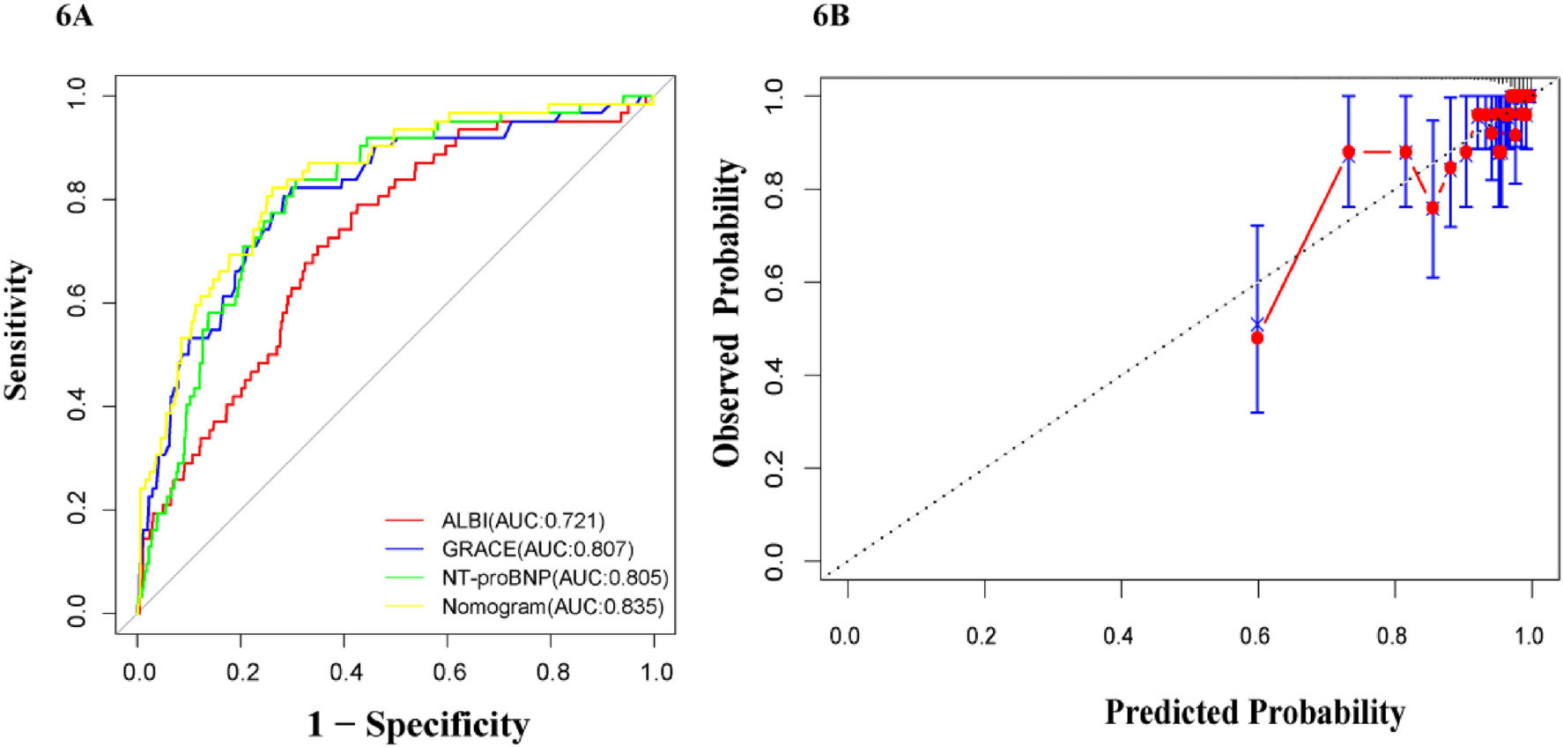
ROC of different models **(A)**, and calibration curve of the nomogram.

AUC were used to examine the discriminative ability of our Nomogram, ALBI, GRACE and NT-proBNP. As shown in Figure 6, ROC curve analysis showed that our Nomogram model performed best. The AUC for our nomogram, ALBI, GRACE, or NT-proBNP was 0.835, 0.72, 0.807, and 0.805, respectively. The DeLong test suggested a statistically significant difference between the new model and ALBI, GRACE, or NT-proBNP in the ability to differentiate patients with death (All *P* < 0.05). To further evaluate the improvement of ALBI on GRACE scores, we calculated the Net Receiver Operating Characteristic Improvement (NRI) and Integrated Discrimination Improvement (IDI) to quantify the incremental value of incorporating ALBI into the original model. The analysis yielded an IDI value of 0.030 (95% CI: 0.002–0.069, *P* < 0.001) and an NRI value of 0.200 (95% CI:: 0.008–0.266, *P* < 0.001). Both metrics indicate a significant enhancement in risk classification capability. And The calibration plots demonstrate good calibration for the models (Figure 6B).

## Discussion

The present study, data from a real-world registry, aimed to assess the potential of combining ALBI with GRACE for improving risk stratification for NSTEMI patients. The main conclusions were as follows: (1) Elevated ALBI levels were significantly associated with a greater risk of all-cause death in NSTEMI patients; (2) RCS analysis indicates that ALBI was positively correlated with all-cause mortality risk in NSTEMI patients. ROC analysis demonstrates its strong predictive capability for mortality risk in NSTEMI patients.; and (3) Adding ALBI and NT-proBNP to GRACE score models can significantly enhance the ability to predict all-cause death outcomes.

Oxidative stress, inflammation, and nutritional status are important pathophysiological mechanisms of AMI, closely related to its occurrence and progression. Previous studies have confirmed that factors such as white blood cells, neutrophils (19), TNF-α, IL-6 (20), and C-reactive protein (21) are closely associated with the severity of AMI and long-term prognosis, gradually becoming important markers for evaluating the progression of AMI. Serum albumin is a marker of inflammation and nutrition (22). As a major component of plasma, albumin has long been considered an important indicator of nutritional status. However, increasing evidence suggests that albumin levels can reflect the degree of systemic inflammation, organ (e.g., heart, kidney, liver) function, and the severity of many diseases (23). Studies have shown that low serum albumin levels are associated with poor prognosis in AMI and chronic coronary artery disease (24). In AMI patients, reduced serum albumin at admission is linked to in-hospital outcomes, long-term survival in myocardial infarction patients, and no-reflow after primary percutaneous intervention. Yoshioka et al. (25) conducted a retrospective study, and the results showed that the cumulative incidence of the main composite events was higher in the group with hypoalbuminemia. After adjusting for relevant clinical variables, hypoalbuminemia remained an independent predictor. Some researchers also conducted cross-sectional studies, and the results showed that a lower serum albumin concentration was associated with the first occurrence of acute myocardial infarction (26). In a study following 2,305 first-onset AMI patients and found that low serum albumin levels at admission were independent predictors of long-term all-cause mortality, cardiovascular mortality, and cardiac mortality in these patients (27). Our study also indicates that decreased albumin levels increase the risk of out-of-hospital mortality in myocardial infarction patients, which is consistent with previous research findings.

Bilirubin, as an endogenous antioxidant, exhibits antioxidant, anti-inflammatory, and cytoprotective effects (28). The bilirubin can suppress nonspecific inflammation, reducing the production of IL-1β, TNF-α, and IFN-γ, and prevent the oxidation of low-density lipoprotein cholesterol (LDL-C), thereby mitigating systemic inflammation and oxidative stress damage (29). Munire et al. (30) studied 615 STEMI patients undergoing PCI and found that the bilirubin level was higher in the MACE group (17.21 ± 6.49 mmol/L vs. 13.34 ± 4.91 mmol/L, *P* < 0.001), and the post-PCI TIMI flow grade was lower (*P* = 0.023). There are scholars observed that the high-bilirubin group had significantly higher in-hospital mortality and MACE rates (*P* < 0.001) (31). A comprehensive analysis identified that the high-bilirubin group had a higher incidence of MACE (16.9% vs. 5.6%, *P* < 0.001) and cardiac mortality (15.3% vs. 4.5%, *P* < 0.001) compared to the low-bilirubin group (32). Elevated bilirubin levels may reflect chronic inflammation and increased oxidative stress. However, some scholars have reached different conclusions in their research. A follow-up study of 4,151 STEMI patients undergoing PPCI found that the high-TBil group had significantly lower MACE rates than the low-TBil group (3.5% vs. 11.0%, *P* = 0.001) (33). Wei et al. reported that MACE patients had lower bilirubin levels (18.3 ± 6.7 vs. 14.8 ± 6.6 mg/L, *P* < 0.01) (34). The findings are inconsistent, and our results also did not reveal significant abnormalities, suggesting that bilirubin alone may not be a robust predictor of AMI prognosis.

The ALBI score is calculated from serum albumin and total bilirubin, although extreme hyperbilirubinemia is directly toxic and has prognostic significance in advanced disease, mild elevations may reflect adaptive, potentially protective metabolic processes when liver function is preserved. The ALBI score provides a more stable and integrated indicator of overall pathological burden by combining bilirubin with albumin. This score captures elements of acute and chronic injury (via bilirubin) while reflecting concomitant functional reserve (via albumin), providing a more robust composite measure of inflammation, oxidative stress, and nutritional status than any single marker. Studies have shown that the ALBI score can serve as a prognostic tool for heart disease. Previous research has confirmed the correlation between the ALBI score and in-hospital mortality in patients with dilated cardiomyopathy (14). Patients with higher ALBI grades had significantly lower cumulative long-term survival rates (log-rank = 45.50, *P* < 0.001), and the ALBI score was independently associated with long-term mortality. Several studies report that the ALBI score is a reliable independent predictor of mortality in patients with hyper-trophic cardiomyopathy, heart failure (35). Based on these findings, we aimed to explore the relationship between the ALBI score and out-of-hospital mortality in patients with acute myocardial infarction (AMI). Using various analytical methods, including univariate analysis, multivariate adjustment, and model fitting, our results indicated that the ALBI score is a risk factor for out-of-hospital death in AMI patients, whether treated as a continuous variable [HR: 3.42 (95% CI: 1.66–7.03), *P* < 0.001] or a categorical variable (HR = 3.43, 95% CI: 1.05–11.22, *P* = 0.041). An elevated ALBI score in AMI patients is closely associated with an increased risk of all-cause mortality. A higher ALBI score is a strong independent predictor of AMI related death (AUC > 0.70).

The GRACE score was currently widely used for prognostic assessment in NSTEMI, but its predictive efficacy is limited, possibly due to the lack of inclusion of newer indicators related to heart failure, inflammation, and nutrition. Japanese scholars have improved the prediction value of in-hospital mortality in acute myocardial infarction patients undergoing PCI through a novel risk stratification system called the “Angiographic GRACE Score”, which adjusts the GRACE risk score based on the culprit coronary artery and its blood flow information (36). Some scholars also combined N-terminal NT-proBNP with the traditional GRACE scoring system and found that the modified GRACE scoring system (AUC = 0.809, *P* < 0.001) was significantly superior to the traditional GRACE scoring system (AUC = 0.786, *P* < 0.001) (12). Based on previous research findings, incorporating indicators such as inflammation and immunity into the GRACE score also appears to enhance its predictive performance. Lu et al. (37) studied 1,357 patients diagnosed with NSTEMI and validated that biomarkers improved the predictive accuracy of the GRACE score by calculating the C-index, net reclassification improvement (NRI), and integrated discrimination improvement (IDI). The results showed that combining NT-proBNP, fibrinogen, and D-dimer increased the prognostic value of GRACE for MACE. A retrospective study of 175 patients with myocardial infarction showed that the AUC value of the Glasgow Prognostic Score combined with GRACE and platelet-lymphocyte ratio was 0.841 (95% CI: 0.761–0.921) (38). Considering the convenience of testing indicators, we combine with ALBI, a comprehensive index reflecting inflammation and oxidative stress, into the GRACE score and obtained favorable results. Similarly, combining ALBI with the GRACE score better predicted mortality in acute myocardial infarction patients. Therefore, we further combined the ALBI score with BNP and found through ROC curve analysis that BNP and the GRACE score could reasonably predict mortality risk, with AUC values of 0.807 and 0.805, respectively. Additionally, combining ALBI further improved predictive accuracy (AUC = 0.835). To facilitate clinical application, we developed Nomogram model based on these three factors, allowing clinicians to input relevant data to generate patient risk assessments.

In practice, the ALBI score is an easily accessible and non-invasive indicator. It can be integrated into routine clinical assessments to rapidly evaluate mortality risk for patients with conditions such as acute myocardial infarction, complementing existing tools like the GRACE score. The newly developed nomogram model integrates the ALBI score with other key predictive indicators, thereby enabling more personalized and quantitative risk stratification. This model can inform multiple critical clinical decisions, including identifying high-risk patients who require more intensive monitoring and closer follow-up, and guiding decisions about more aggressive or personalized treatment strategies in the early stages of the disease. However, we should also recognize that its clinical utility must be validated through prospective studies involving larger, multicenter, and racially diverse populations.

## Limitations

This study has several limitations. First, due to the constraints of the retrospective design, we were unable to dynamically measure bilirubin and albumin levels during patient follow-up. Additionally, the patients were from China, lacking diversity in geographic regions and racial backgrounds. Second, as a single-center study with a limited sample size, even after adjusting for multiple confounding factors, some data bias remained unavoidable. Despite using multivariable Cox regression to adjust for these differences, residual confounding due to unmeasured factors or the inherent limitations of statistical adjustment for the observed imbalances (particularly in age, NT-proBNP, LVEF, and key medications) may persist and could influence the observed associations. Additionally, due to the low frequency of death events, this may increase the risk of overfitting. Third, limited clinical data prevented examination of prognostic differences among acute myocardial infarction (AMI) patients based on the ALBI score and various treatment strategies. Thus, prospective cohort studies are needed to validate our findings.

## Conclusion

The ALBI score strongly correlates with mortality in patients with NSTEMI, and our new Nomogram, based on the ALBI, NT-proBNP, and GRACE scores, shows good discriminative ability to predict the risk of mortality in patients with NSTEMI.

## Data Availability

The raw data supporting the conclusions of this article will be made available by the authors, without undue reservation.
