# Supplementary material for: Predictive value of ALBI score in assessing prognosis of NSTEMI: a retrospective cohort study
Source: Front Cardiovasc Med. 2026 Apr 22;13:1768037. doi: 10.3389/fcvm.2026.1768037 (PMC13143790; doi:10.3389/fcvm.2026.1768037)
Supplement: Supplementary file 1 [file Table1.docx]

Supplementary Material

**Predictive Value of ALBI Score in Assessing Prognosis of NSTEMI: A Retrospective Cohort Study**

Xiaoqiang Chen^†^, Xintao Zhou^†^, Chuanglu Zhao, Lishan Chen, Shuyin Wang, Xinwen Min, Jishun Chen^*^, Xiaolei Li^*^

Sinopharm Dongfeng General Hospital (Hubei Research Center of Hypertension), Hubei University of Medicine,Shiyan,Hubei, 442000, China

^†^ Xiaoqiang Chen and Xintao Zhou contributed equally to this work.

*Correspondence: Xiaolei Li, E-mail: [lixiaoleishz@163.com](mailto:lixiaoleishz@163.com); Jishun Chen, E-mail: [376370276@qq.com](mailto:myt-xj@163.com)

**
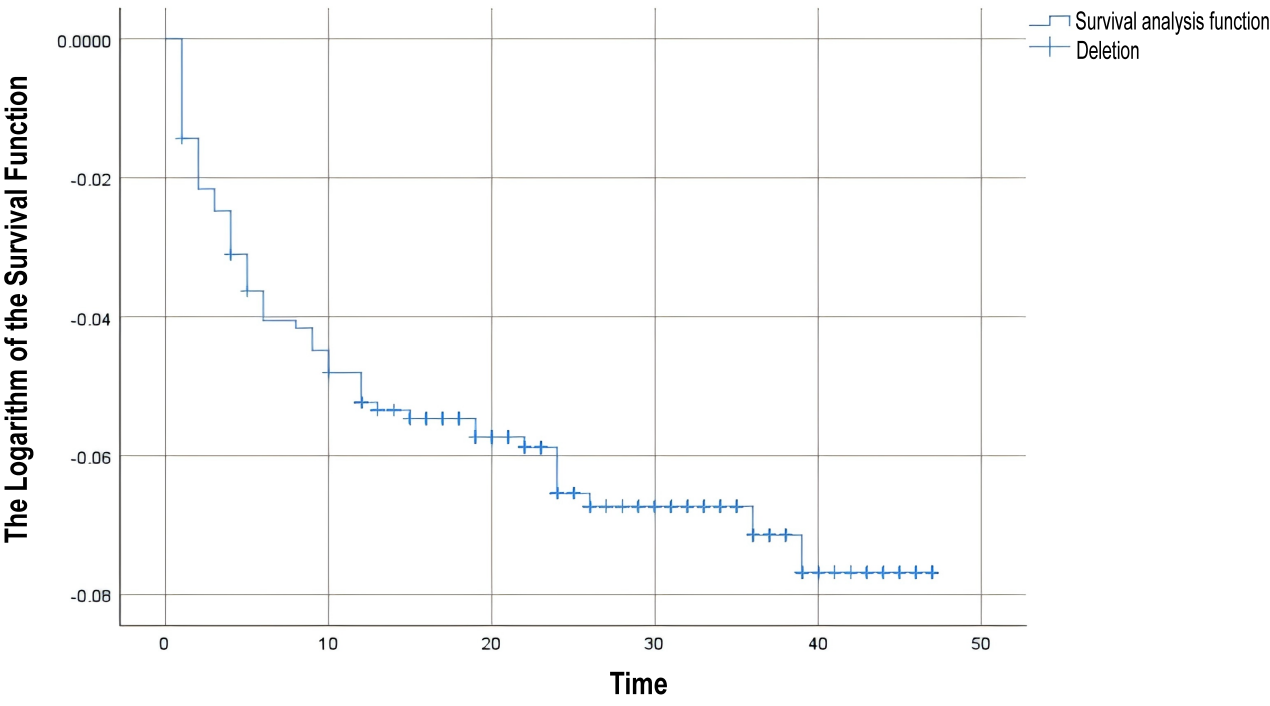
**

Fig.S1 Kaplan-Meier survival analysis curve

Table S1. Cox Regression Analysis of ALBI Score for Predicting Out-of-Hospital Mortality

| Variables | Model | |
| --- | --- | --- |
|  | HR (95%CI) | *P* |
| ALBI(continuous) | 5.09 (2.86-9.08) | <0.001 |
| ALBI (categorical) |  |  |
| Q1 (≤ -2.60) | Reference |  |
| Q2(-2.60 to -2.36) | 2.08 (0.55-7.88) | 0.282 |
| Q3(-2.36 to -2.08) | 4.86 (1.43-16.49) | 0.011 |
| Q4 (> -2.08) | 6.81 (2.05-22.65) | 0.002 |

| HR: Hazard Ratio, CI: Confidence Interval |
| --- |
| The model accounted for variables such as ALBI, age, sex, and NT-proBNP. |
